# Nutshell Materials as a Potential Eco-Friendly Biosorbent for the Effective Extraction of UV Filters and Parabens from Water Samples

**DOI:** 10.3390/ma17205128

**Published:** 2024-10-21

**Authors:** Izabela Narloch, Grażyna Wejnerowska, Przemysław Kosobucki

**Affiliations:** Department of Food Analysis and Environmental Protection, Faculty of Chemical Technology and Engineering, Bydgoszcz University of Science and Technology, 85-326 Bydgoszcz, Poland; izabela.narloch@pbs.edu.pl (I.N.); p.kosobucki@pbs.edu.pl (P.K.)

**Keywords:** benzophenones, biosorbent, emerging contaminants, extraction method, nutshells, parabens, UV filters

## Abstract

UV filters and parabens, as ingredients of cosmetics, are commonly occurring water pollutants. In our work, nutshells were used as biosorbents in the developed analytical procedure for the determination of UV filters and parabens in water samples. The shells obtained from walnuts, hazelnuts, peanuts and pistachios were applied as biosorbents. The proposed analytical method can be used as a powerful alternative to other methods for the analysis of UV filters and parabens in water samples. A method of carrying out the sorption step and its parameters, i.e., the effect of time, pH, and salt addition, was developed. A method for the desorption of analytes was also developed, in which the type and volume of solvent, and the desorption time, were established. The recoveries were in the range of 59–117% for benzophenones and lower recoveries from 14 to 75% for parabens. The results showed that nutshells can be used as low-cost, efficient and eco-friendly biosorbents for the determination of parabens and UV filters in water samples. These materials can be used as a ‘greener’ replacement for the commercially available adsorbents for the extraction of cosmetic ingredients from the environment.

## 1. Introduction

Due to the fact that UV filters and paraben preservatives are widely used components in many personal care products, they are commonly found in the environment. These substances are an increasingly serious environmental problem, due to their increasing detection in waters and marine biota (e.g., in fish, mollusks, and corals). Depending on the season, location, public access and sampling conditions, the detectability of UV filters and parabens in water ranges from ng/L to mg/L [1]. Growing concerns about the possible harmful effects (e.g., endocrine-disrupting, carcinogenic, neurotoxic, and bioaccumultive) of environmental pollutants have led to an increasing amount of research to determine their ecological and physiological effects [2,3]. Due to the possibility of continuously exposing humans to these chemicals, it is important to monitor and strictly control the water environment in terms of the content of UV filters and parabens in it.

For this purpose, extraction techniques play an important role in sample preparation for the determination of UV filters and parabens in environmental water samples. According to the literature, the most commonly used extraction techniques are liquid–liquid extraction (LLE) and solid-phase extraction (SPE). However, these techniques, due to the consumption of large volumes of samples and organic solvents, as well as high time consumption, have been replaced by microextraction techniques. These techniques include solid-phase microextraction (SPME), stir-bar sorptive extraction (SBSE), dispersive solid-phase extraction (dSPE), microextraction by packed sorbent (MEPS), bar adsorptive microextraction (BAµE), and dispersive liquid–liquid microextraction (DLLME) [4,5,6]. They are characterized by high efficiency, speed, and low costs, as well as the possibility of extensive modification by introducing new materials and solvents.

In recent years, analytical chemistry has been striving to develop analytical procedures based on the principles of Green Analytical Chemistry (GAC) and its development White Analytical Chemistry (WAC). Therefore, both economically and environmentally, attention is paid to the reduction, reuse and recycling of materials used in analytical methods that do not negatively affect analytical performance [7]. For this reason, biosorbents are promising materials in extraction techniques due to their high extraction capacity, non-toxicity, low cost, biodegradability and environmental friendliness [8,9]. Biosorbents are divided into three main groups: microorganisms (fungi, bacteria and algae), chitin/chitosan and lignocellulose. Among the groups mentioned, the most frequently used biosorbent is lignocellulose, which is isolated from plant tissues or obtained from unrefined plant products, such as tree barks or corks [10]. One example of biosorbents consisting of lignocellulose are nutshells. The literature contains studies using these sorbents to remove metals [11,12,13,14], pesticides [15,16], dyes [17,18], and pharmaceuticals [19,20,21]. However, no studies were found regarding the use of nutshells as sorbents used for the determination of personal care products, i.e., parabens and sunscreen filters in water samples.

The aim of this study was the development of an analytical methodology for determining the amounts of UV filters (benzophenones) and parabens in water matrices. For this purpose, for the first time, the walnut, hazelnut, peanut and pistachio shells were applied as sorbents for the analytes. The proposed method is eco-friendly, low-cost, and fast. The optimization, validation, and application of the proposed analytical method to water matrices are fully discussed.

## 2. Materials and Methods

### 2.1. Materials and Reagents

Analytical standards of methyl paraben (MP), ethyl paraben (EP), propyl paraben (PP), and butyl paraben (BP) were purchased from Sigma-Aldrich (Darmstadt, Germany), while benzophenone (BPZ), benzophenone-1 (BP1), benzophenone-3 (BP3), benzophenone-8 (BP8) and decane, used as internal standard (IS), were obtained from Sigma-Aldrich Co. (St. Louis, MO, USA). The structures and relevant physicochemical properties of analytes are exhibited in Table 1. These standards were used to prepare a 1 mg/mL stock solution in methanol (MeOH). The stock solution was used to prepare a working solution with a concentration of 0.5 mg/L of each analyte in ultrapure water. The hydrochloric acid (36%), which was used for pH adjustment, and the salting-out effect were evaluated with the addition of sodium chloride (NaCl) obtained from Chempur (Piekary Śląskie, Poland). For re-dissolving analytes, EA/ACN mixture (1:1, *v:v*) with IS at a concentration of 10 µL/L was used. Ethyl acetate (EA), acetonitrile (ACN) and MeOH were supplied from Merck (Darmstadt, Germany) and were analytical grade.

The water sample was collected on June from a lake (Pieczyska beach, Koronowo, Poland) and stored in sealed bottles at 4 °C in their raw form without filtration, for a few days, until the moment of the analysis.

### 2.2. Instrumentation

Chromatographic analyses were performed using an Agilent 7890B (Agilent, Santa Clara, CA, USA), equipped with a split/splitless injector, multipurpose autosampler, and flame ionization detector (FID).

The GC was fitted with a ZB-5 column (Zebron, Phenomenex Inc., Torrance, CA, USA), 30 m × 0.25 mm × 0.25 µm, containing (5% phenyl)-methylpolysiloxane.

The injector port was held at 230 °C and used in the split mode using a split ratio of 5:1, and the injection volumes were 1 µL. The detector temperature was 250 °C. The GC oven temperature program started at 80 °C and increased to 240 °C at 8 °C/min, where it was held for 13 min.

The structural characterization of the nutshells was performed by using a Bruker ALPHA Fourier-transform infrared spectrophotometer (FT-IR) (Berlin, Germany), using an attenuated total reflection technique (4500–360 cm^−1^ wavelength range). The morphology of the biosorbent was evaluated by scanning electron microscopy (SEM) using a LEO Electron Microscopy Ltd. 1430 VP (Cambridge, UK).

### 2.3. Preparation of the Biosorbent Material

Walnuts, hazelnuts, peanuts and pistachios were obtained from supermarket, Bydgoszcz, Poland. Firstly, the shells were separated from the nuts, and the materials were washed abundantly with tap water at room temperature. The wet shells were placed in a laboratory dryer for 12 h at 80 °C. Then, the nutshells were ground (Polymix PX MFC 90D, Kinematica, Switzerland) to obtain a particle size of 800–500 µm (~55%) and <500 µm (~45%). Part of the material was ground (Grindomix GM 200, Retsch, Germany) to smaller particles of 500–200 µm (~50%) and <200 µm (~50%). Lastly, 5 g of prepared shell powder was placed in a beaker, and the material was washed with hot water (~80 °C) until colorless water was obtained. Next, the shells were washed with 5 mL of EA, and placed in a laboratory dryer for 12 h at 80 °C. The prepared biosorbents were stored in closed dark bottles.

### 2.4. Extraction Procedure

**Figure 1:**
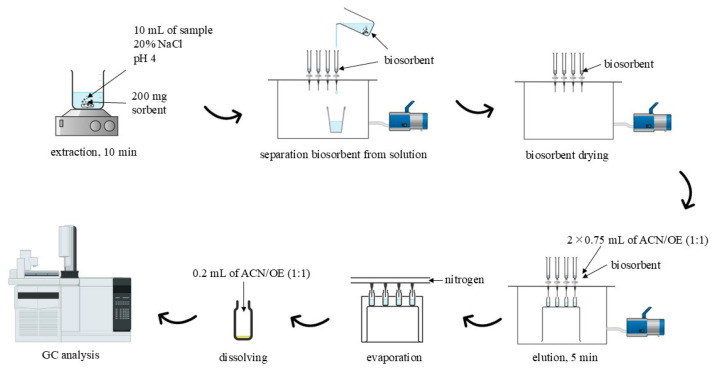
Scheme of the proposed method.

The extraction procedure is depicted in Figure 1. The extraction step was performed in a beaker containing 200 mg of biosorbent, and 10 mL of sample adjusted at pH 4 and 20% *w*/*v* of NaCl for 10 min. After that, the biosorbent was separated from the solution using empty SPE cartridges and SPE vacuum manifold. The biosorbent was dried by air (5 min). Then, material was immersed in the 750 µL of ACN/OE mixture (1:1, *v*:*v*) for 2.5 min, and the desorption step was repeated. The obtained extract was subjected to by nitrogen steam until evaporation (15 min). Then, 0.2 mL of ACN/OE mixture (1:1, *v*:*v*) containing IS was added to solution residue, and the extract was subjected to the GC analysis.

### 2.5. Method Validation

The analyte relative recovery and the intra- and inter-day precisions were determined by the analysis of lake samples spiked at three concentrations: 50, 200, and 500 µg/L. Precision was calculated as the relative standard deviation (RSD), considering a precision of less than 20% as the acceptance criterion. Repeatability was assessed by performing three determinations in one day for each concentration level. Intermediate precision was assessed by three determinations on another day for the medium concentration level (200 µg/L). Accuracy was evaluated as the percentage of recovery considering an acceptance criterion of 60–120% [22,23]. The experiments were performed in triplicate and the analysis was repeated at least three times.

## 3. Results and Discussion

### 3.1. Characterization of Biosorbents

**Figure 2:**
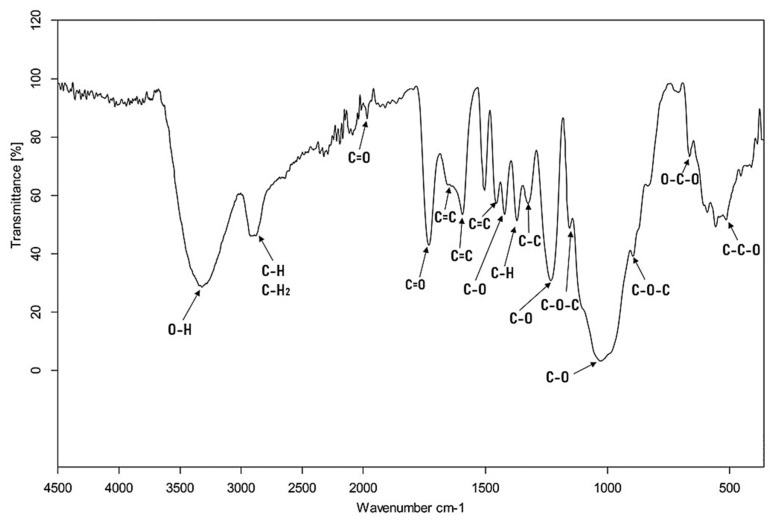
FT-IR spectrum of the walnut shells.

Fourier transform infrared (FT-IR) spectroscopy was used to identify the main functional groups present on the four biosorbents’ surfaces. Due to the fact that the obtained spectra are similar to each other, only the spectrum of the walnut shell is shown in Figure 2. All tested materials showed common peaks associated with the main components present in nutshells: cellulose, hemicellulose, and lignin. An O-H stretching broad band at 3318 cm^−1^ and two sharpened bands at 2913 and 2879 cm^−1^ were observed in the C-H and C-H_2_ stretching region—these bands are assigned to the methyl and methylene groups from lignin, hemicellulose, and cellulose. The intense C=O stretching band at 1731 cm^−1^ corresponds to the acetyl and ester groups in hemicellulose. The peak observed at 1654 and 1593 cm^−1^ is associate with the C=C group in aromatic groups of hemicellulose and lignin. The aromatic regions at 1504 and 1455 cm^−1^ correspond to lignin. The absorption peak at 1370 cm^−1^ is associated with the C-H group from the lignin methoxy groups, while C-C aromatic bonds are verified at 1326 cm^−1^. The peaks at 1231 and 1156 cm^−1^ correspond to the C-O, and C-O-C stretching vibrations. The peak observed at 1028 cm^−1^ include contributions from lignin methoxy groups, cellulose, and hemicellulose ester groups. The infrared spectrum obtained is very similar to other spectra observed for nutshells in the literature [11,13,24].

**Figure 3:**
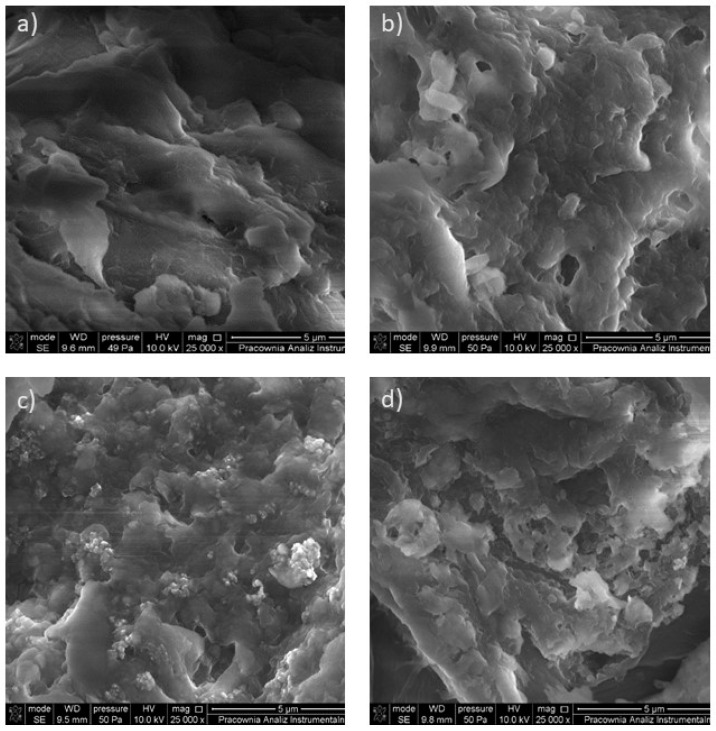
SEM images of (**a**) walnut shells; (**b**) walnut shells after adsorption of analytes; (**c**) peanut shells; (**d**) peanut shells after adsorption of analytes.

Scanning electron microscopy (SEM) was used to study the morphology of biosorbents, and the exemplary SEM micrographs before and after the sorption process using/for walnut and peanut shells are shown in Figure 3. The adsorbents exhibit irregular, rough, and porous structures of various shapes and sizes. Such structures are characteristic of lignocellulosic sorbents. As can be seen in Figure 3b,d after the sorption process, the sorbent structure became thicker and more folded. It may indicate the physical adsorption through adhesion of the analytes in the pores and on the surface of the biosorbent.

### 3.2. Preliminary Research

Methods of preparing an aqueous sample for chromatographic analyses using a biosorbent as a loose sorption bed are known from the literature. They consist, among others, of placing the sorbent in the pipette tip [25], and placing the sorbent in an empty SPE column [26]. After performing test analyses using these methods for the extraction of parabens and benzophenones, satisfactory results were not obtained. Expecting better extraction efficiency, it was decided to extend the contact time of the biosorbent with the analytes (the proposed method). As expected, the results at the initial stage were much better. The proposed procedure for sample preparation (extraction and desorption, shown in Figure 1) required the optimization of the sorption and desorption stages.

Preliminary studies were also conducted to determine the method of purifying the biosorbent. Different amounts of hot water (100–500 mL) and solvent (EA) (1–10 mL) were used for this purpose. Satisfactory results were obtained using 250 mL of hot water and 5 mL of EA for the Optimization of the proposed procedure.

### 3.3. Optimization of the Proposed Procedure

Several factors affecting the extraction efficiencies of the proposed method were tested, including the amount of biosorbent, the pH of the solution, the salting-out effect, the extraction time, the kind and volume of desorption solvent, the number of desorption cycles, and the desorption time. A mixed benzophenones and parabens standard containing 500 µg/L of each analyte was used to examine the extraction efficiency of the method. Walnut shells as biosorbents with a particle size of 800–500 µm (~55%) and <500 µm (~45%) were used for the optimization studies. The optimized parameters of the analytical method obtained using walnut shells were applied to determine the extraction efficiency for other shells. All optimization experiments were carried out in triplicate (n = 3).

#### 3.3.1. Optimization of Biosorbent Mass

**Figure 4:**
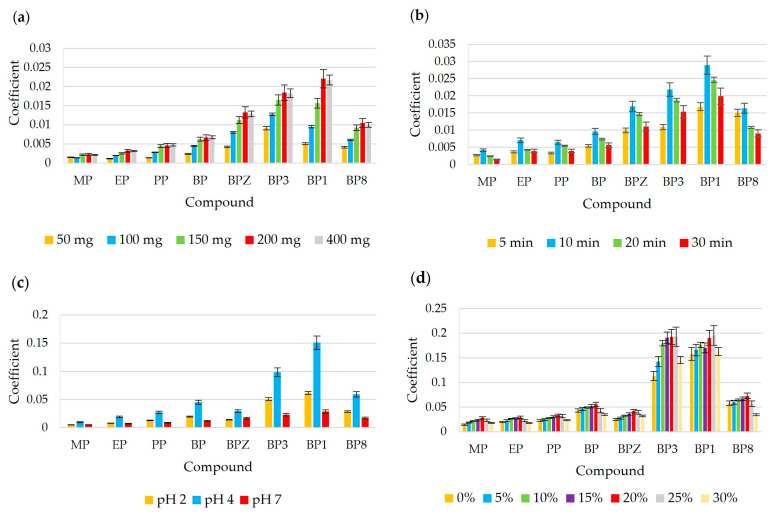
A bar graph for the optimization of (**a**) biosorbent mass: 10 mL of sample, pH 7, 20 min; (**b**) extraction time: 10 mL of sample, pH 7, biosorbent mass 200 mg; (**c**) pH solution: 10 mL of sample, 10 min, biosorbent mass 200 mg; (**d**) salt addition: 10 mL of sample, pH 4, 10 min, biosorbent mass 200 mg. For all optimization tests, the desorption step was performed with 1 × 1500 µL of ACN for 15 min.

In order to obtain the highest possible amounts of extracted analytes, different masses of biosorbent ranging from 50 to 400 mg were evaluated for the extraction of the tested analytes. As can be seen in Figure 4a, the best results were obtained using the biosorbent at a dose of 200 and more mg. Therefore, 200 mg of the sorbent was selected for further tests.

#### 3.3.2. Optimization of Extraction Time

As expected, it was shown that extraction time is an important parameter which influences the effectiveness of the analytes extracted. The effect of extraction time was evaluated in the range from 5 to 30 min. Figure 4b illustrates the effect of extraction time on the coefficient of the analytes. The extraction efficiency increased with increasing time, up to 10 min, and thence began to decrease. This could be due to the redissolution of the analyte into the sample solution. These results show that 10 min is enough for the complete equilibration of the analytes and biosorbent.

#### 3.3.3. Optimization of pH

The pH value is important because it affects the ionization state and solubility of analytes in water. In this experiment, different sample solutions containing benzophenones and parabens with varying pH, namely 2, 4, and 7. The highest extraction efficiency for the proposed method was achieved at pH 4, where an approximately 100% increase in coefficient was achieved with respect to pH 7. For the efficient extraction of benzophenones and parabens (pK_a_ ≥ 7), the pH of the sample solution should be lower than the pK_a_ of the analytes in order to obtain the target analytes in non-ionized forms, so that they have a greater tendency to partition into the organic phase. The results are summarized in Figure 4c.

#### 3.3.4. Optimization of Salting-Out Effect

The ionic strength adjustment by the salt addition was an also important parameter that could affect the extraction of the analytes. In the case of polar analytes (log K_ow_ < 4), the addition of NaCl, which increases the ionic strength, causes the hydration of salt ions, making water less accessible to organic compounds, promoting their migration towards the sorbent phase and reducing the solubility of analytes. The effect of ionic strength was studied by the addition of various amounts of NaCl (in the range from 0 to 30% *w*/*v*) to sample solutions. The extraction efficiency increases with increasing NaCl from 0 to 20%, and then decreases (Figure 4d). A decrease in salt contents greater than 20% may be due to an increase in solution viscosity, which in turn reduces the extraction kinetics. Therefore, the 20% addition NaCl to the sample solution was chosen as the optimal amount.

#### 3.3.5. Optimization of the Desorption Step

**Figure 5:**
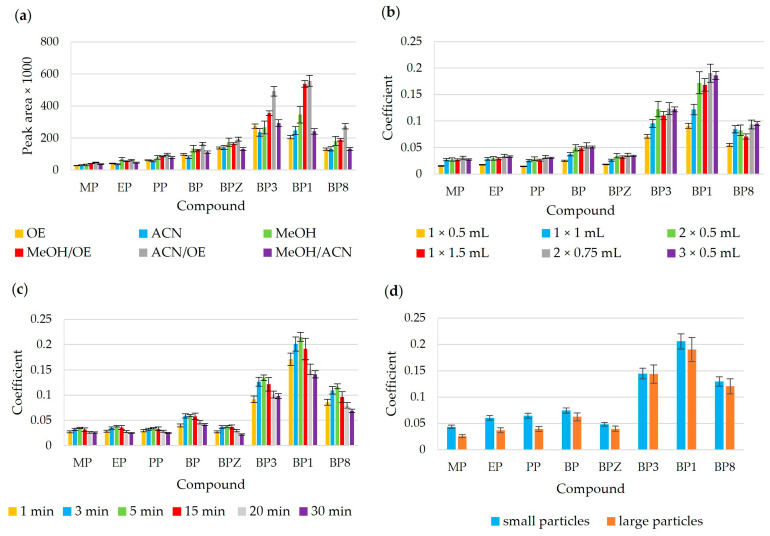
A bar graph for the optimization of the desorption condition: (**a**) the kind of desorption solvent: 1 × 1500 µL of solvent, 15 min; (**b**) the volume of solvent and number of cycles: desorption with ACN/OE, 15 min; (**c**) desorption time: 2 × 750 µL of ACN/OE; (**d**) biosorbent size: desorption with 2 × 750 µL of ACN/OE, 5 min. For all optimization tests, the sorption step was performed with 200 mg of biosorbent and 10 mL of sample, pH 4, 20% NaCl for 10 min.

The next relevant step of the optimization method was assigning the conditions of the desorption. Due to the fact that the tested analytes are polar (log K_o/w_ < 4), a polar solvent was used for desorption of the analytes. Based on previous experiences and literature data, a polar solvent was selected for desorption, containing methanol (MeOH; log K_o/w_ = −0.77), ethyl acetate (OE; log K_o/w_ = 0.73), and acetonitrile (ACN; log K_o/w_ = −0.34). The highest extraction efficiency, especially for BP1, BP3 and BP8, was achieved when mixture of ACN/OE (1:1, *v*:*v*) was used as the desorption solvent. Thus, it was selected as the optimal desorption solvent (Figure 5a). Moreover, the volume and number of cycles of desorption solvent (ACN/OE mixture) was studied. For this purpose, 0.5, 1.0 and 1.5 mL of desorption solvent (ACN/OE mixture) was used. In order to be able to compare the results, the obtained coefficients were correlated with each other. It can be seen that using cyclic desorption gives better results for both the 1000 µL and 1500 µL volumes. The best response was obtained using 1.5 mL (2 timesfor 750 µL), and it was selected as the optimum volume for the desorption solvent (Figure 5b). In addition, desorption times of 1, 3, 5, 15, 20, and 30 min were evaluated and the best results was achieved with 5 min (Figure 5c).

#### 3.3.6. Optimization of the Biosorbent Size

The last step of the optimization tests was to check how the size of biosorbent particles affects the extraction of analytes. For this purpose, two sizes of particles of nutshells were used: small particles (500–200 µm (~50%) and <200 µm (~50%)) and large particles (800–500 µm (~55%) and <500 µm (~45%)). Based on the obtained results, it can be concluded that more effective extraction was obtained using smaller biosorbent particles for all tested shells. For the walnut, hazelnut and peanut shells, smaller particles performed better than larger particles by 1 to 19%. For the pistachio shells, smaller particles gave better results by 1 to 40%, including 1–17% for benzophenones and 17–40% for parabens, as shown in Figure 5d. Smaller particles absorb analytes better because of their greater access to active sites on external surfaces and within pores.

### 3.4. Analytical Figures of Merit

The accuracy and repeatability of the proposed method using the nutshells as sorbent were evaluated by spiking the real samples (lake water sample) with analytes at concentration levels of 50, 200, and 500 µg/L. The recoveries ranged from 14 to 75% for parabens and from 59 to 117% for benzophenones (Table 2). It can be seen that in the developed method satisfactory recoveries (>60%) were obtained only for benzophenones. Particularly high recoveries (≥70%) was obtained for BP1, BP3, and BP8. The highest recovery rates for benzophenones were obtained using peanut shells (84–117%) and pistachio shells (87–101%). While, the RSDs of the procedure were satisfactory for both groups of analytes and the repeatability was lower than 20%.

The enrichment factors (EFs), calculated from the ratio of the extracted analyte concentration in the solvent phase to the initial concentration in the aqueous phase, were found to be in the range of 7–37.5 for parabens and 19.5–58.5 for benzophenones.

The proposed method was applied in the determination of parabens and benzophenones in lake water samples and the concentrations of the analytes were all below the quantification limits (LOQ: 30–90 µg/L for parabens and 15–33 µg/L for benzophenones).

### 3.5. Greenness Assessment

**Figure 6:**
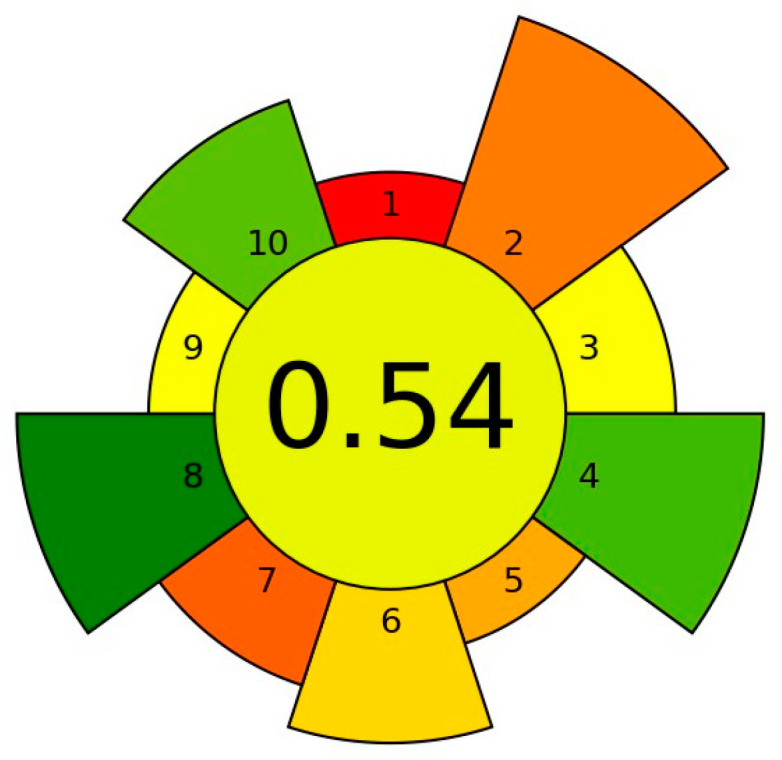
An assessment of greenness of the proposed sample preparation method, obtained by using the AGREEprep tool.

The proposed sample preparation method was evaluated using the Analytical Greenness metric for sample preparation (AGREEprep); the metric was introduced in 2022 by Wojnowski et al. [27]. In the AGREEprep metrics, the sample preparation method is assigned a score related to using the solvents, materials and reagents, waste generation, energy consumption, sample amount, and throughput. Each part has a score of 0–1, and the proximity to 1 indicates the greenness of the method. A method that achieves a total score greater than 0.5 is classified as a “green” method. The pictogram shown in Figure 6 was obtained as a result of using the AGREEprep metric tool software, evaluating the ten assessed categories and the total assessment, which is 0.54. A summary of the aspects considered in each category is detailed in the generated report (Supplementary Material) with calculated score values.

The lowest scores were obtained in categories 1 and 7, which concerned (principle 1) the location of sample preparation (the need to perform tests in a laboratory) and the aspects of “*integration and automation*” (principle 7)—the procedure requires three steps (extraction, elution and evaporation). Nevertheless, the greenest advantages of the proposed method were related to the low waste generation (principle 4), estimated as 0.4 mL per sample, the low energy consumption (3 Wh per sample, principle 8), and the “*operator’s safety*” (principle 10), which only involves one hazard related to the consumption of ACN and OE as solvents. Taking into account the obtained evaluation results, it can be stated that the proposed method can be classified as a “green” sample preparation method.

### 3.6. Comparison Proposed Method with Other Methods

The efficiency of proposed procedure was evaluated by comparing it with other methods [25,26,28,29,30,31] which use biosorbents for the determination of UV filters and parabens in water samples (Table 3). Among the mentioned methods, the bar adsorptive microextraction (BAµE) was most frequently used; however, in comparison to the method proposed by us, it uses a larger sample volume, and the extraction of analytes takes much longer (>45 min). In general, when comparing the developed method with other methods, a similar analytical performance was obtained in relation to precision and recoveries. Additionally, the sensitivity of the proposed method may be lower when a different detector (e.g., MS, MS/MS) is used.

## 4. Conclusions

Nutshells are one of the wastes produced by the food industry and have been proven to be an economical substitute sorbent for the identification and quantification of benzophenones and parabens in water. The developed analytical method, for the first time, uses nutshells such as hazelnut, walnut, peanut and pistachio shells to determine the above-mentioned analytes in a water matrix. Nutshells have proven to be an effective natural sorbent that can retain the ingredients of personal care products from the aqueous matrix. According to the principles of green sample preparation, this method is characterized by low organic solvent consumption, low energy consumption, simplicity, speed, miniaturization and the use of a safe, sustainable, renewable and biodegradable sorbent. Therefore, it is environmentally friendly according to the so-called Green Analytical Chemistry, which was confirmed by the AGREEprep tool. Good enrichment factors, high relative recovery and other satisfactory analytical results were obtained, in particular for benzophenones. Studies show that nutshells are an alternative sorption material for commercially available sorbents. However, they require further intensive research on the possibility of their wider use in analytical chemistry.

## Figures and Tables

**Table 1:** The physicochemical properties and structure of the studied compounds.

Compound	Abbreviation	Formula	CAS Number	Chemical Structure	Molecular Weight (g/mol)	Log K_o/w_	pK_a_
Methylparaben	MP	C_8_H_8_O_3_	99-76-3	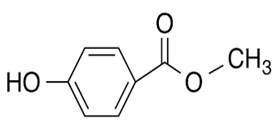	152.15	1.96	8.17
Ethylparaben	EP	C_9_H_10_O_3_	120-47-8	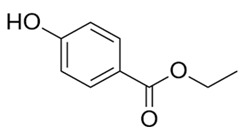	166.17	2.47	8.22
Propylparaben	PP	C_10_H_12_O_3_	94-13-3	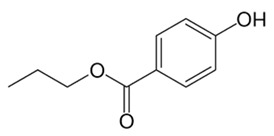	180.21	3.04	8.35
Butylparaben	BP	C_11_H_14_O_3_	94-26-8	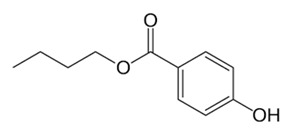	194.23	3.57	8.37
Benzophenone	BPZ	C_13_H_10_O	119-61-9	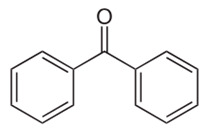	182.22	3.18	7.5
Benzophenone-1	BP1	C_13_H_10_O_3_	131-56-6	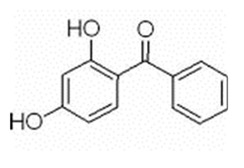	214.22	3.15	7.53
Benzophenone-3	BP3	C_14_H_12_O_3_	131-57-7	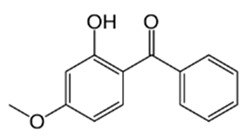	228.24	3.79	7.56
Benzophenone-8	BP8	C_14_H_12_O_4_	131-53-3	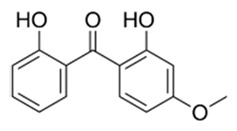	244.24	3.82	6.99

Data were obtained from: https://pubchem.ncbi.nlm.nih.gov (accessed on 31 July 2024).

**Table 2:** Analyte relative recoveries and intra- and inter-day precision in the lake samples (n = 3).

Analyte	Spiked Concentration (µg/L)	Walnut Shells			Hazelnut Shells			Peanut Shells			Pistachio Shells		
		RR (%)	RSD, Intra-Day (%)	RSD, Inter-Day (%)	RR (%)	RSD, Intra-Day (%)	RSD, Inter-Day (%)	RR (%)	RSD, Intra-Day (%)	RSD, Inter-Day (%)	RR (%)	RSD, Intra-Day (%)	RSD, Inter-Day (%)
MP	50	23	14.7	15.1	25	11.6	12.8	26	14.3	11.6	28	15.6	15.9
	200	16	15.6	18.9	18	11.2	17.4	14	9.7	12.6	16	18.6	14.6
	500	20	18.2	13.1	20	16.9	14.2	33	20.0	17.6	32	11.3	13.6
EP	50	28	11.2	12.8	26	12.3	11.2	53	9.6	11.3	49	12.9	12.3
	200	16	10.3	12.3	19	9.6	10.5	40	14.5	9.7	39	9.0	12.1
	500	33	16.1	11.0	28	18.4	17.4	52	12.3	14.3	46	14.7	9.6
PP	50	36	13.3	14.8	34	11.6	12.6	58	8.9	10.2	61	16.3	11.2
	200	20	9.8	12.6	27	8.3	9.6	41	4.3	7.3	57	4.0	6.9
	500	35	13.3	9.0	42	14.7	11.7	60	10.6	12.3	57	16.9	10.3
BP	50	49	8.2	11.8	48	11.6	18.5	69	16.3	17.6	67	14.0	11.3
	200	37	17.1	16.7	42	7.8	9.6	57	13.2	14.9	55	12.3	10.3
	500	53	14.3	12.8	47	14.2	11.2	75	11.8	12.8	73	8.6	9.3
BPZ	50	41	12.6	11.9	48	16.9	13.6	60	6.9	7.3	61	7.9	9.8
	200	49	15.7	18.3	52	12.4	10.5	66	3.5	9.8	68	12.3	9.8
	500	50	14.7	12.7	51	11.6	11.9	64	11.3	10.3	65	15.6	11.2
BP3	50	91	16.9	14.6	90	9.4	10.8	98	9.1	11.2	96	14.9	10.3
	200	87	2.9	9.3	86	10.3	13.6	84	19.3	17.6	91	10.2	9.8
	500	86	11.6	13.9	81	12.3	14.9	117	15.6	14.3	95	9.6	9.9
BP1	50	76	15.9	12.8	73	16.7	15.9	110	13.6	11.2	101	11.3	10.6
	200	69	0.8	7.9	69	15.6	13.8	102	7.8	10.9	95	8.7	9.3
	500	69	14.7	12.7	67	6.8	9.3	108	12.3	10.3	89	12.6	11.0
BP8	50	84	5.9	8.9	88	11.2	10.8	105	16.9	15.6	94	15.7	14.7
	200	80	16.4	14.8	82	14.3	16.9	101	12.6	14.9	87	5.0	9.0
	500	73	11.2	9.6	90	9.8	10.4	102	11.3	13.6	88	16.3	15.2

**Table 3:** Comparison of proposed method with other methods reported in the literature.

Analytes ^a^	Biomaterial	Matrix	Technique ^b^	Instrumentation	Sample Volume	Solvent for Desorption (Volume)	Extraction Time	Recovery (%)	Ref.
MP, EP, PP, BP	cork	river water	BAµE	HPLC-DAD	15 mL	MeOH and ACN (120 μL)	45 min	53–124	[28]
MP, EP, BPZ	diatomaceous earth	lake water	BAµE	HPLC-DAD	15 mL	MeOH and ACN (100 μL)	90 min	63–124	[29]
MP, PP, BP, BPZ	*Araucaria angustifolia* bracts	river water	BAµE	HPLC-DAD	30 mL	ACN and water (80 µL)	180 min	62–115	[30]
MP, EP, BPZ, 4-MBC, OD-PABA	cork	lake water	DPX	HPLC-DAD	800 µL	MeOH and ACN (100 μL)	90 s	71–132	[25]
ES, EDP, IMC, OCR, EMC, 4-MBC, HS	cellulose	spiked water	disk-SPE	HPLC-UV-Vis	20–100 mL	2-propanol (4500 µL)	-	60–70	[26]
4-MBC, OD-PABA	cork	river water	SPME	GC-MS	25 mL	-	70 min	67–107	[31]
MP, EP, PP, BP	walnut, hazelnut, peanut, pistachio shells	lake water	-	GC-FID	10 mL	ACN and OE (1500 µL)	10 min	14–75	this work
BPZ, BP1, BP3, BP8								59–117	

^a^ OD-PABA: 2-ethylhexyl 4-(dimethylamino)benzoate; 4-MBC: 3-(4-methylbenzyli-dene)camphor; OCR: octocrylene; EMC: 2-ethylhexyl 4-methoxycinnamate; MBC: 3-(4-methylbenzyli-dene)camphor; HS: homosalate; ES: 2-Ethylhexyl salicylate; EDP: 2-ethylhexyl 4-(dimethylamino)benzoate (ethylhexyl dimethyl PABA); IMC: isoamyl 4-methoxycinnamate. ^b^ DPX: disposable pipette extraction; SPME: solid-phase microextraction; BAµE: bar adsorptive microextraction.

## Data Availability

The original contributions presented in the study are included in the article and Supplementary Material, further inquiries can be directed to the corresponding author.

## Supplementary Materials

The following supporting information can be downloaded at: https://www.mdpi.com/article/10.3390/ma17205128/s1, Supplementary Material S1: AGREEprep report.
