# Supplementary material for: Nutshell Materials as a Potential Eco-Friendly Biosorbent for the Effective Extraction of UV Filters and Parabens from Water Samples
Source: Materials (Basel). 2024 Oct 21;17(20):5128. doi: 10.3390/ma17205128 (PMC11509166; doi:10.3390/ma17205128)
Supplement: Supplementary file 1 [file materials-17-05128-s001.zip › materials-3183248-supplementary.pdf]

Nutshell Materials as a Potential Eco-Friendly Biosorbent for the Effective Extraction of UV Filters and Parabens from Water Samples

Izabela Narloch , Gra˙zyna Wejnerowska * and Przemysław Kosobucki

AGREEprep

Analytical Greenness Metric
for Sample Preparation

* evaluation for simultaneous preparation of 6 samples

09/08/2024 09:32:44

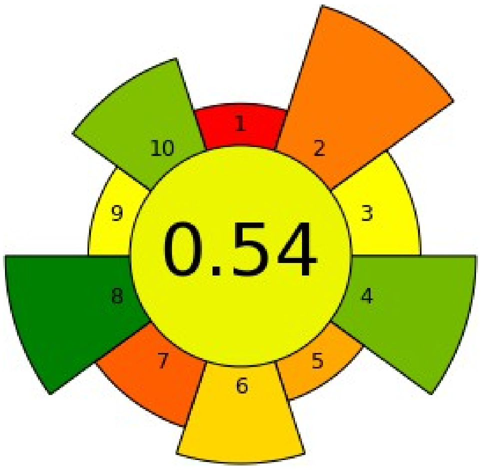

#	Criterion	Score	Weight
1.	**Sample preparation placement**<br>Sample preparation placement: Ex situ	0.0	1
2.	**Hazardous materials**<br>Mass [g] or volume [mL] of problematic materials: 1.9	0.24	5
3.	**Sustainability and renewability of materials**<br>50-75% of reagents and materials are sustainable or renewable and can only be used once	0.5	2
4.	**Waste**<br>Mass [g] or volume [mL] of waste: 0.4	0.78	4
5.	**Size economy of the sample**<br>Mass [g] or volume [mL] of the sample: 10	0.33	1
6.	**Sample throughput**<br>Hourly sample throughput: 6	0.42	3
7.	**Integration and automation**<br>No. of sample prep. steps: 3 steps; degree if automation: Manual systems	0.19	2
8.	**Energy consumption**<br>Approximate energy consumption per analysis [W]: 3	1.0	4
9.	**Post-sample preparation configuration for analysis**<br>GC with non-MS detection, atomic absorption spectroscopy, capillary electrophoresis, etc.	0.5	1
10.	**Operator's safety**<br>No. of distinct hazards: 1 hazard	0.75	3
